# Comments on Regulation of paraquat for wheat crop contamination by Gupta, S. et al., DOI https://doi.org/10.1007/s11356-022-20816-8

**DOI:** 10.1007/s11356-023-25823-x

**Published:** 2023-03-11

**Authors:** Steve Crook, Caroline Willetts

**Affiliations:** Syngenta Ltd, Jealott’s Hill International Research Centre, Jealott’s Hill, Warfield, Bracknell, RG42 6EY UK

Dear Editor,

We are writing to you with reference to the paper published online in *Environmental Science and Pollution Research, * Regulation of paraquat for wheat crop contamination by Gupta, S. *et al*. https://doi.org/10.1007/s11356-022-20816-8. The study was of considerable interest to us as we take seriously any suggestion that crop residues of our products may be a human health concern.

Having examined the paper, we believe that issues with the analytical method have led to the misidentification of high residues in wheat grain, leading to the erroneous hypothesis that paraquat can be translocated from the soil into wheat grain.

The significant flaws identified in the paper’s analytical method were:

- The retention time of paraquat is given as 1.4 min which implies that paraquat was not retained or very poorly retained on the selected C18 column.
- The calibration curves presented in the paper are exponential. In our experience when using UV detection for the analysis of paraquat, a linear response over several orders of magnitude is obtained.
- Paraquat is prone to adsorb to glass and metal surfaces and plastic vessels should be used for paraquat analysis. If calibration standards were prepared in glass vessels, paraquat would be lost from the solution. This is more pronounced at lower concentrations and could be a cause of the exponential calibration curves and the high residues seen. There is no mention in the paper of the types of vessels used for analysis.
- The extraction method used was a cold shake with methanol. To extract genuinely incurred paraquat residue from solid matrices requires harsh extraction methods, e.g. hours of reflux with a strong acid solution.
- No confirmatory analysis was presented. With the minimal retention reported, residues in control samples and residue levels that appear to significantly exceed the permitted national MRL, it would have been appropriate to confirm the presence of paraquat by a more selective technique, e.g. LC–MS/MS.

The abstract of the paper states that “Paraquat can pose potential health hazards if it is translocated from soil into wheat grains, but no study is available for its possible translocation causing wheat grain contamination”. There is a wealth of independently reviewed scientific evidence to show that paraquat is not translocated from the soil to wheat grain, e.g. the 2004 JMPR/Codex paraquat evaluation. In addition, the review has details of the validated analytical methods for paraquat. https://www.fao.org/fileadmin/templates/agphome/documents/Pests_Pesticides/JMPR/Evaluation04/paraquat.pdf PARAQUAT (057) accessed June 2022.

Additionally, Syngenta has conducted long-term field trials in Australia, UK and the USA using highly exaggerated field application rates on plots in which wheat was grown, and these have demonstrated no detectable residues in wheat grain and that residues of paraquat do not move from the soil into wheat grain. Paraquat reaching the soil is extremely strongly adsorbed, biologically deactivated and unavailable to plants. (Roberts et al. 2002**,** Deactivation of the Biological Activity of Paraquat in the Soil Environment: A Review of Long-Term Environmental Fate. *J. Agric. Food Chem 50,* 3623–3631).

Paraquat is registered in both the USA and Australia as a pre-emergence herbicide. Both of these countries have independent pesticide monitoring programmes and to our knowledge paraquat has not been detected at or above MRLs in wheat grain or processed wheat commodities in either country. https://www.agriculture.gov.au/sites/default/files/documents/wheat-residue-testing-datasets-2020-21.pdf National Residue Survey, Department of Agriculture, Water and the Environment, accessed June 2022.

The clear conclusion, consistent with the data from crop rotation studies and global MRLs, is that pre-emergence uses of paraquat cannot and do not result in detectable residues of paraquat in wheat grain. The data presented in the paper is inconsistent with our current knowledge base for paraquat and we would very much like to understand the reasons for this. We would welcome any comments or additional observations from the paper’s authors with respect to the points raised above.

Please do not hesitate to contact us if you need any clarification or additional information.

With Kind Regards,

Steve Crook,  Principal Technical Expert, Metabolism and Analytics, Syngenta Ltd.

Caroline Willetts, Senior Technical Expert, Product Safety, Syngenta Ltd.
